# Aberrant Lipid Metabolism Promotes Prostate Cancer: Role in Cell Survival under Hypoxia and Extracellular Vesicles Biogenesis

**DOI:** 10.3390/ijms17071061

**Published:** 2016-07-02

**Authors:** Gagan Deep, Isabel R. Schlaepfer

**Affiliations:** 1 Department of Cancer Biology, Wake Forest Baptist Medical Center, Winston-Salem, NC 27157, USA; gdeep@wakehealth.edu; 2 Division of Medical Oncology, Genitourinary Cancer Program, University of Colorado School of Medicine, Aurora, CO 80045, USA

**Keywords:** prostate cancer, lipogenesis, fatty acid oxidation, carnitine palmitoyltransferase (CPT1), hypoxia, re-oxygenation, extracellular vesicles

## Abstract

Prostate cancer (PCa) is the leading malignancy among men in United States. Recent studies have focused on the identification of novel metabolic characteristics of PCa, aimed at devising better preventive and therapeutic approaches. PCa cells have revealed unique metabolic features such as higher expression of several enzymes associated with de novo lipogenesis, fatty acid up-take and β-oxidation. This aberrant lipid metabolism has been reported to be important for PCa growth, hormone-refractory progression and treatment resistance. Furthermore, PCa cells effectively use lipid metabolism under adverse environmental conditions for their survival advantage. Specifically, hypoxic cancer cells accumulate higher amount of lipids through a combination of metabolic alterations including high glutamine and fatty acid uptake, as well as decreased fatty acid oxidation. These stored lipids serve to protect cancer cells from oxidative and endoplasmic reticulum stress, and play important roles in fueling cancer cell proliferation following re-oxygenation. Lastly, cellular lipids have also been implicated in extracellular vesicle biogenesis, which play a vital role in intercellular communication. Overall, the new understanding of lipid metabolism in recent years has offered several novel targets to better target and manage clinical PCa.

## 1. Introduction

Lipids are a large and diverse class of biomolecules of unique chemical structure in terms of fatty acid chain length, number and location of double bonds and backbone structures (like glycerol for triglycerides). The functional consequence of this lipid diversity still remains unknown, however, lipids are known to exert multiple biochemical functions including fuel for cancer growth, metastasis and resistance to therapies. Historically, lipids have been viewed as passive components of plasma membranes, facilitating signaling cascade recruitment and promoting signal transduction. Using tandem mass spectrometry (MS/MS) and Raman scattering microscopy [1], we now know that lipid content and composition can markedly alter the behavior of cancer cells. For example, changes in lipid composition (saturated vs. unsaturated fatty acids) can alter membrane fluidity, cell growth and resistance to chemotherapeutic drugs [2,3]. Especially, lipid metabolism plays a key role in cancer cell survival and even proliferation under stressful hypoxic conditions. Furthermore, besides modification of growth factor receptors at the plasma membrane, lipids can also lead to the generation of bioactive molecules and extracellular vesicles, enhancing the communication between cells and the tumor microenvironment [4]. Thus, how cancer cells handle their cellular lipids is extremely diverse and dynamic, offering possibilities for therapeutic interventions that could complement current anti-cancer therapies.

In this review, we have discussed the role of aberrant lipid metabolism in prostate cancer, including the importance of lipid accumulation and lipid metabolites in the survival and extracellular vesicle biogenesis of hypoxic cancer cells. We have mostly covered studies in prostate cancer (PCa) field but wherever pertinent, we have described studies in other cancers as well.

## 2. Aberrant Lipid Metabolism in PCa (Prostate Cancer)

### 2.1. De Novo Lipid Synthesis Is a Characteristic of Cancer Cells

The Warburg effect (a high glycolysis rate under normoxic conditions) is a characteristic of cancer cells that has been known for decades [5]. However, cancer cells also show changes in lipid synthesis, uptake and metabolism. In the adult mammal, de novo synthesis of fatty acids is restricted mainly to liver and adipose tissues, while the majority of the lipid demand of other tissues is fulfilled by circulating lipids in blood. However, early studies in tissue slices from tumors showed that cancer cells could incorporate radioactively-labeled glucose into lipids, underscoring the fact that tumors could make their own lipids using sugar carbons [6]. Further evidence for de novo lipogenesis in tumors came from the observation that fatty acid synthase (FASN) is overexpressed in several cancers, including breast [7] and PCa [8]. These observations have important clinical implications, since combinatorial therapies that target lipid synthesis and growth could be more effective for steroid-responsive tumors which are known to accumulate lipid, like PCa and breast cancers. In fact, the positive effect of steroids on lipid accumulation in cancer cells may help explain increased cancer risk in individuals who received hormone replacement therapy [9,10,11].

PCa is a hormone-dependent cancer that grows slowly and is mainly dependent on lipid oxidation for fuel. This is probably the reason why positron emission tomography (PET) does not work well in PCa, since the tumors do not show a strong avidity for the radioactive fluorodeoxyglucose (FDG) label [12,13]. PCa is the most commonly diagnosed non-cutaneous malignancy and the second highest contributor to cancer deaths after lung cancer in men in the United States [14]. Currently, the standard systemic treatment for advanced PCa is based on androgen deprivation with initial positive responses, but PCa tumors eventually become resistant through several pathways, including de novo androgen biosynthesis [15]. After PCa becomes castration-resistant no curative treatments exist, making the identification of novel therapies imperative. The way castration-resistant tumors activate lipid metabolism is unknown but likely involves a gene expression program orchestrated by novel and restored androgen-receptor (AR) mediated signaling pathways [16]. In fact, lipid synthesis via FASN is a major target of androgen action in PCa cells [8,17], and it is associated with cell growth, survival and drug resistance [17,18]. Interestingly, the role of lipogenesis regulators (SREBP1A/1C) has also reported in PCa progression to androgen-independence [19]. However, the identification of lipogenic enzymes as targets for therapy remains challenging, with many lipid-related drugs under study for cancer treatment [20].

**Figure 1:**
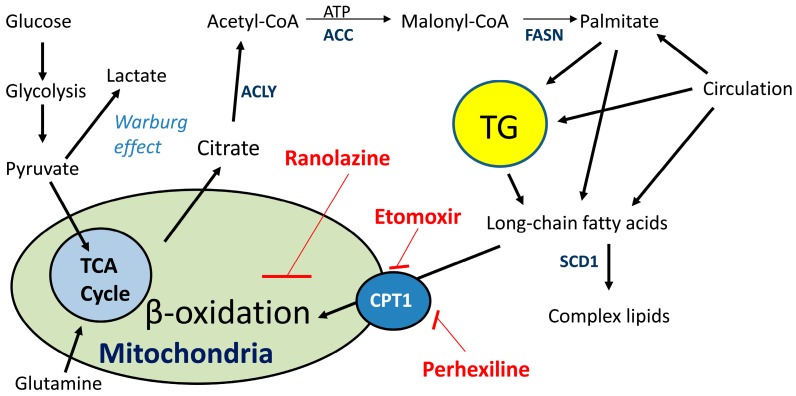
Warburg effect, lipid metabolism and site of action for metabolic drugs. Fatty acids are available from the circulation (diet), de novo synthesis from glucose and glutamine carbons or from hydrolysis of TG stores in lipid droplets. Fatty acids enter the mitochondria via the rate-limiting transporter CPT1. The metabolic drugs etomoxir and perhexiline inhibit entry of fatty acids into mitochondria by irreversibly inhibiting CPT1. Ranolazine works by decreasing β-oxidation in the mitochondrial matrix. The Warburg effect is active in the presence of oxygen, when lipids can be oxidized in the mitochondria. During hypoxia, the Warburg effect is enhanced as well as de novo lipogenesis, with a concomitant reduction in lipid oxidation, resulting in lipid accumulation inside the cells. ACC: acetyl-CoA carboxylase, SCD1: steroyl-CoA-desaturase, ACLY: ATP citrate lyase, CPT1: carnitine palmitoyl-transferase, TG: triglyceride, FASN: fatty acid synthase, TCA: tricarboxylic acid.

Overexpression of key enzymes in lipid synthesis in PCa is characteristic of both primary and advanced disease [21], suggesting that targeting lipid metabolism enzymes in PCa may offer new avenues for therapeutic approaches. Candidate-gene expression studies have identified genes involved in lipogenesis (Figure 1), which are essential for development and progression of a wide variety of cancers. In addition to FASN, increased expression of other lipogenic enzymes, such as acetyl-CoA carboxylase (ACC), steroyl-CoA-desaturase (SCD1) and ATP citrate lyase (ACLY) represent a nearly-universal phenotypic alteration in many tumors [22]. In particular, SCD1 is a microsomal enzyme that catalyzes the committed step in the biosynthesis of the monounsaturated fatty acids (MUFA) from saturated fatty acids (SFA) by introducing a *cis*-double bond to the acyl chain. The preferred substrates are palmitate (16:0) and stearate (18:0), which yield palmitoleate (16:1n-7) and oleate (18:1n-9), respectively. These represent the major MUFA of membrane phospholipids, triglycerides, wax esters, and cholesterol esters. In fact, most cancer cells contain higher levels of MUFA that tend to partition into detergent-resistant lipid rafts [2,23]. Because the desaturation index (ratio of MUFA to SFAs) affects phospholipid composition, and alteration in this ratio has been observed in several cancers, targeting SCD1 is therapeutic target in hormone dependent cancers. Inhibition of SCD1 in cancer cells blocks lipid synthesis, decreases growth and viability, making it an ideal target for therapeutic intervention [24,25]. Particularly, inhibition of SCD1 resulted in decreased prostate tumor growth in mice xenografts following pharmacologic treatment with a small inhibitor molecule [2].

### 2.2. Lipid Oxidation Supports Cancer Growth and Survival

Although research in cancer lipid metabolism has been focused on de novo lipogenesis, recent studies have clearly shown that lipid catabolism is important in cancer cell survival, for example, monoacylglycerol lipase which catalyzes the release of fatty acids from intracellular lipid stores, promotes tumor growth and survival [26]; blocking fat oxidation results in significant death of leukemia cells exposed to pro-apoptotic agents [27]; fatty acid oxidation increases resistance to radiation and chemotherapeutic agents [28]; and finally, fatty acid oxidation fuels the production of metabolites needed to synthesize lipids and to protect cells from oxidative stress [29].

Candidate gene expression and gene copy number alterations analysis have identified upregulated transcripts involved in lipid catabolism of malignant tumors [30,31,32,33,34]. Among them, the rate-limiting enzymes for fat oxidation carnitine palmitoyltransferase 1 isoforms A and C (CPT1A and C) are overexpressed in many human tumors [35,36]. Increased expression of CPT1C, induced by AMPK and p53, has been shown to protect cancer cells from death when exposed to low glucose and hypoxic conditions. On the other hand, knockdown (KD) of *CPT1* sensitizes cancer cells to radiation and anti-cancer drugs, by inducing metabolic stress leading to apoptosis [27,37,38]. Furthermore, *CPT1* genes are also critical for tumor progression and metastasis, resulting in the activation of oncogenes that promote metastasis [33]. Altogether, lipid oxidation is an important component of cancer metabolism together with aerobic glycolysis and lipogenesis, but still remains ill-defined in cancer metabolism [39].

Currently, PCa patients that fail to respond to treatment with anti-androgens (like enzalutamide) have no curative therapy and this represents a subset of resistant tumors that show small-cell or neuroendocrine features on metastatic biopsy [40]. Interestingly, examination of the c-BioPortal database (available at: http://www.cbioportal.org) for gene amplification and mRNA data, shows that the *CPT1A* gene is amplified in 22% of cases (*n* = 107) in the neuro-endocrine prostate cancer dataset from the Trento/Cornell/Broad 2016 group [30]. This study brings attention to the drug-resistant PCa tumors, including LNCaP cells that were treated for a long time with enzalutamide in the lab, mimicking the drug resistance phenotype observed in the clinic. The fact that they find 22% of their cases with increased CPT1A alteration underscores the possibility of a metabolic treatment that could target this neuro-endocrine and castration-resistant PCa that is presently incurable. Additionally, another important analysis of the stand-up-2-cancer group (SU2C/PCF Dream team) showed *CPT1A* gene alteration (mainly amplification) in 11% of PCa cases (*n* = 150) [41]. Altogether, genetic databases reveal an important role for β-oxidation in the progression and drug resistance underpinnings of PCa.

One way to study β-oxidation in a translational research manner is using safe metabolic inhibitors that can be used in the lab and the clinic. Several lipid catabolism inhibitors are now available that show low toxicity and could be implemented in the clinic swiftly: Etomoxir, perhexiline and ranolazine, (Figure 1). Fatty acids (from diet or from triglyceride (TG) breakdown) can be used for fuel (via β-oxidation) and promote growth, or used to generate lipid signaling molecules that shape the fate of the cell, like eicosanoids [42,43] and phospholipids [44].

Etomoxir is a safe irreversible inhibitor of the long chain fatty acid transporter and has been used in the treatment of heart failure [45]. Etomoxir works by inhibiting CPT1 and blocking the entry of long chain fatty acids into the mitochondria for oxidation, forcing cells to use the oxidation of glucose for energy [12,46,47]. Ranolazine is an FDA-approved drug known to reduce β-oxidation in the heart but their mechanism of action is not as well defined as etomoxir [48]. Ranolazine is considered a partial β-oxidation inhibitor and it has shown anti-cancer effects in leukemia and breast cancer. Another drug targeting CPT1 and safe for human use is perhexiline [49], which has shown a renaissance in its use in both refractory angina and chronic heart failure, potentially opening doors for its use in cancer therapy. For a comprehensive list of lipid-related drugs for cancer treatment see [20].

Presently, there are no studies of these metabolic drug effects on prostate lipid metabolism and androgen action, making them attractive tools to explore the mechanisms of lipid use in prostate cancer and design targeted therapies. Blockade of lipid oxidation via CPT1 has been shown to lead to metabolic stress, mainly due to decrease ATP production and increased apoptosis in human colon cancer cells [36]. As expected, this phenotype was associated with accumulation of lipid droplets likely due to the inability to burn the lipid in the mitochondria. One of the consequences of toxic lipid accumulation is the development of endoplasmic reticulum (ER) stress, which is an alarm mechanism to try to restore the normal function of the ER, that is, the synthesis and processing of secretory and membrane proteins [50]. Failure in the attempts to restore ER homeostasis usually result in cell death, including cancer [51,52,53]. Studies from fatty-liver disease have clearly shown that delivery of long chain saturated fatty acids to liver cells or increasing the amount of saturated fatty acids (like palmitate) within the liver provokes ER stress, apoptosis and liver injury [54,55,56]. These studies have led to the hypothesis that the catabolism of fatty acids delivered to or stored within cells is an important determinant of ER homeostasis, apoptosis and disease progression.

Our work over the recent years with PCa cells and lipid metabolism has shown that interruption of lipid oxidation in the mitochondria via CPT1 results in a momentary increase in glucose uptake [12] that ultimately leads to apoptosis, ER stress, increased ceramide production and decreased androgen action [38]. These studies have led to the hypothesis that the ability to burn lipid in the mitochondria is an important determinant of lipid homeostasis and PCa progression. Furthermore, the ability of the cells to burn lipid is directly associated with their survival to common tumor microenvironment changes like hypoxia-reoxygenation patterns typical of solid tumors, including PCa [4].

## 3. Hypoxia and Lipid Metabolism in PCa

### 3.1. Role of Hypoxia in PCa

Hypoxia refers to a decreased O_2_ level in various parts of a tumor especially towards the core due to limited oxygen diffusion and/or abnormal tumor vasculature. Hypoxia is an early event during carcinogenesis and it is considered an important biomarker for disease aggressiveness [57,58,59,60,61]. Compared with normal prostate epithelium, hypoxia-inducible factor-1α (HIF1α) is over expressed in primary PCa [62]. Importantly, there are numerous studies suggesting that HIF1α could be an important biomarker to predict PCa progression and treatment outcomes [59,63]. In this regard, Ranasinghe et al. reported that HIF1α expression in PCa is an independent risk factor for disease progression associated with significantly reduced metastasis-free survival and CRPC (castrate-resistant prostate cancer)-free survival [59]. The study by Vergis et al. also concluded that higher HIF1α expression in PCa patients treated with radiotherapy was a significant predictor of early biochemical failure [60].

Milosevic et al. conducted one of the largest clinical studies characterizing PCa hypoxia in 247 patients and directly measured the oxygen level in tumors by using ultrasound-guided trans-rectal needle-electrode [57]. This study concluded that hypoxia in primary tumors is associated with early biochemical relapse after radiotherapy and predicted local recurrence. In another study, nonspecific HIF1α inhibitors (digoxin, metformin and angiotensin-2 receptor blocker) increased the progression-free survival and decreased the risk of developing metastasis and CRPC in PCa patients on androgen deprivation therapy [64]. Platz et al. also concluded that digoxin use had a 25% lower risk of PCa [65]. All these clinical studies suggested that hypoxia is an important determinant of disease progression and treatment outcomes in PCa.

### 3.2. Hypoxia-Inducible Factor (HIF) and Lipid Mediators Exert a Positive Feedback Loop in Hypoxic PCa Cells

HIF, a transcriptional factor, is one of the major mediators of cellular adaptation to hypoxia via regulation of genes that control several features of cancer pathogenesis such as aberrant metabolism, neo-angiogenesis, epithelial-to-mesenchymal transition (EMT), increased stemness and therapeutic resistance [66,67,68]. HIF belongs to the large family of basic-helix–loop–helix proteins and is a heterodimer of one of three oxygen-regulated HIFα subunits (HIF1α, HIF2α and HIF3α) and a stable HIF1β subunit. Under normoxia, prolyl hydroxylases (PHD1, 2 and 3) hydroxylate the site-specific proline residues of HIF1α in the presence of oxygen, which is then recognized by an E3 ligase von Hippel-Lindau (VHL), which tags HIF1α for proteasome degradation. Under hypoxia, PHD activity is inhibited, resulting in lesser HIF1α prolyl hydroxylation, which in turn leads to decreased rates of HIF1α polyubiquitination and degradation. Therefore, under hypoxic conditions, HIF1α is stabilized, translocates to the nucleus, dimerizes with HIF1β and binds to hypoxia response elements within the promoters of its target genes [69,70]. We have recently reviewed the role of various signaling pathways (PI3K/AKT/mTOR, NADPH oxidase, Wnt/β-catenin, etc.) in regulating HIF expression under hypoxic conditions [71].

**Figure 2:**
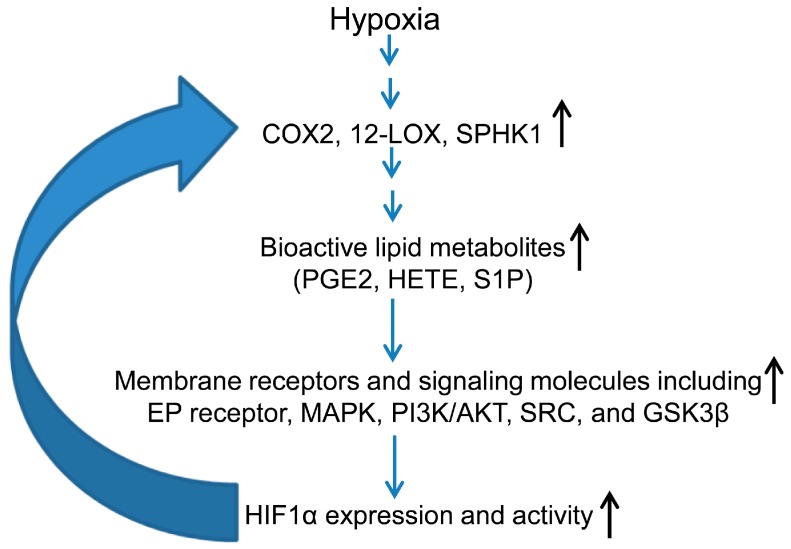
Proposed positive feed-back loop of lipid mediators and HIF1α signaling in hypoxic prostate cancer (PCa) cells. Hypoxic environments induce the activation of lipid enzymes (COX2, LOX, and SPHK1) that generate bioactive lipid molecules that regulate signaling cascades, ultimately activating HIF1α and generating a positive feedback loop that sustains growth in a hypoxic environment. COX2: Cyclooxygenase 2, 12-LOX: 12-Lipoxygenase, SPHK1: Sphingosine kinase 1, PGE2: Prostaglandin E2, HETE: Hydroxyeicosatetraenoic acid, S1P: Sphingosine-1-phosphate, EP receptor: Prostaglandin E receptor; black arrows show increase, blue arrows show the sequential pathway.

Besides the conventional pathways mentioned above, several enzymes and lipid mediators are also associated with increased HIF1α activation and nuclear translocation under hypoxic conditions [72,73,74,75,76]. In particular, the enzyme cyclooxygenase 2 (COX2) is activated in PCa cells under hypoxia and promotes HIF1α stabilization and nuclear accumulation through one of its downstream metabolites, prostaglandin E2 (PGE2) [75,76], which leads to HIF1α activation via multiple signaling molecules including Mitogen-activated Protein (MAP) kinases, SRC and PI3K [72,76]. Furthermore, COX2 was also identified as one of the HIF1α target genes [74], thereby creating a positive feedback loop between these two signaling pathways as shown in Figure 2. Similarly, Krishnamoorthy et al. showed that in PCa cells exposed to hypoxia, the 12-lipoxygenase enzyme (12-LOX) up-regulated the HIF1α mRNA and protein expression, through the generation of the eicosanoid 12-HETE (12-hydroxyeicosatetraenoic acid) [73,74]. Hypoxia also activated the sphingosine kinase 1 (SPHK1) enzyme, which is known to convert sphingosine into sphingosine-1-phosphate (S1P), a potent growth signaling molecule in several cancer models [77]. These studies reveal a complex inter-play between various lipid mediators and HIF1α signaling in hypoxic cancer cells, and also suggest that PCa could be targeted through inhibiting this positive feed-back signaling loop.

### 3.3. Hypoxia Promotes Lipid Accumulation in Cancer Cells

Hypoxia plays a major role in the metabolic reprogramming of cancer cells. Under hypoxic conditions, HIF1α stimulates glycolysis through induction of glucose transporters (GLUT) and several glycolytic enzymes (hexokinase 2, phoshofructokinase-1 and lactate dehydrogenase A) and inhibits mitochondrial respiration by inhibiting pyruvate dehydrogenase kinase 1 [78]. In the past, a lot of research has focused on the molecular understanding of the increased glycolysis in cancer cell survival under hypoxic conditions. However, studies in the past decade have firmly established that besides glycolysis, several aspects of lipid metabolism including accumulation of lipid droplets, lipogenesis and β-oxidation, play key roles in survival and adaptation to low oxygen conditions [79,80,81,82].

Several studies have shown that under hypoxic conditions, glutamine undergoes reductive carboxylation and generates citrate for lipid synthesis, while glucose metabolites play a lesser role in the production of citrate [81,82,83]. Indeed, Metallo et al. showed that cancer cells preferentially used glucose carbons for palmitate synthesis under normoxic conditions. However, fatty acid synthesized under hypoxic conditions were primarily synthesized from glutamine carbons mainly through IDH1 (Isocitrate dehydrogenase)-mediated reductive pathway [81]. Furthermore, VHL-deficient renal carcinoma cells preferentially used reductive glutamine metabolism for lipogenesis even when cultured under normal oxygen levels, whereas re-expression of wild-type VHL in these cells resulted in a switch back to oxidative glucose metabolism as the source of carbon for lipid synthesis [81]. This study concluded that reductively metabolizing amino acids for lipid synthesis under hypoxia, allowed cells to conserve glucose to fuel cancer growth. Subsequently, it was shown that HIF1α promoted the ubiquitination and proteolysis of the α-ketoglutarate dehydrogenase (αKGDH) enzyme, enhancing the dependency on glutamine for lipid synthesis under hypoxic conditions [82].

Besides diverting glutamine for lipogenesis, under hypoxic conditions, HIF1α also regulates the expression of fatty acid synthase (FASN) and lipin 1 (LPIN1), which facilitate fatty acid synthesis and lipid storage [84,85]. Huang et al. showed that under hypoxia, HIF1α suppressed acyl-CoA dehydrogenases and fatty acid oxidation to facilitate cancer progression, via a cross-talk between metabolic and signal transduction pathways [80]. This study suggested that hypoxic cancer cells divert lipids for storage to reduce the oxidative stress resulting from fatty acid oxidation. Jiang et al. also confirmed with three-dimensional multimodal molecular imaging and quantification that volume and concentrations of total choline and lipid were significantly increased in hypoxic regions of breast tumor xenografts when compared to normoxic regions [86]. Earlier, Glunde et al. showed that HIF1α enhances choline kinase expression in hypoxic PCa cells resulting in higher phosphocholine and total choline levels [87]. In another study, Kourti et al. reported that casein kinase 1δ (CK1δ) inhibition in hypoxic cancer cells, stimulated cell proliferation through HIF1α and LPIN1 [88]. This group also reported earlier that CK1δ targets Ser247 at the PAS domain of HIF1α, causing reduction of its in vitro affinity for HIF1β and inhibition of its transcriptional activity [89].

Hypoxia also promotes accumulation of cellular lipid droplets through HIF1α-mediated induction of fatty acid binding protein 3 (FABP3) and FABP7, both of which are involved in fatty acid uptake [79]. Furthermore, hypoxia also induces the expression of adipophilin (ADRP), which is required for the formation of lipid droplets’ membranes. Furthermore, recent studies have shown that inhibition of lipid storage reduced the protection against reactive oxygen species (ROS) toxicity, decreased survival of cells subjected to hypoxia-reoxygenation in vitro and strongly impaired tumorigenesis in vivo [79]. We have reported similar findings, where PCa cells accumulated lipids under hypoxia in association with increased HIF1α, ATP-citrate lyase and FASN expression [4]. We also found that PCa cells rapidly used their stored lipids for proliferation following a hypoxia-reoxygenation paradigm. Importantly, hypoxia-reoxygenated PCa cells were extremely sensitive to growth inhibition in the presence of etomoxir or CPT1 knock-down, which significantly compromised their ability to use stored lipids through β-oxidation [4]. Furthermore, the increased invasiveness of hypoxia-reoxygenated PCa cells was compromised in the presence of celecoxib (COX2 inhibitor), emphasizing the role of arachidonic acid metabolites in the invasiveness of these cells. In another study, Qiu et al. showed that HIF2α-dependent lipid storage in clear-cell renal cell carcinoma played an important role in maintaining the integrity of endoplasmic reticulum (ER) as well as in suppressing cytotoxic ER stress [90].

**Figure 3:**
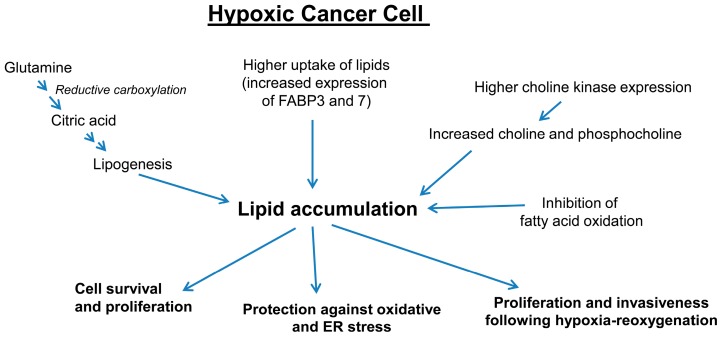
Convergent metabolic pathways in a hypoxic microenvironment; In hypoxic PCa cells, multiple pathways lead to the accumulation of lipids, which play key role in cell survival, protection against stress and proliferation especially following re-oxygenation after hypoxia. FABP, fatty acid binding protein.

Altogether, these studies clearly showed that under hypoxic conditions, PCa cells promote lipid accumulation through de novo lipogenesis mainly through reductive glutamine metabolism, activation of lipogenesis, uptake of fatty acids and via inhibition of fatty acid oxidation (Figure 3). These studies also suggested that accumulated lipids protect the cells from oxidative damage as well as ER stress, and play a key role in fueling the growth and invasiveness of PCa cells following the reoxygenation of hypoxic cells (Figure 3). This hypoxia-reoxygenation effect is very relevant in the therapeutic radiation setting, where the radiation-resistant core of a hypoxic tumor gets exposed to oxygen after the outside tumor layers are killed off. The accumulated lipid in the hypoxic core is then able to use the influx of oxygen to burn lipids and generate ATP and lipid metabolites (via COX or LOX enzymes) to sustain growth.

### 3.4. Role of Hypoxia-Induced Lipid Accumulation in Extracellular Vesicle Biogenesis

Recent studies have firmly established a critical role for nano-sized vesicles in inter-cellular communication in the tumor microenvironment [4,91,92,93,94,95,96]. The role of extracellular vesicles has been reported in primary tumor growth, neo-angiogenesis, pre-metastatic niche formation, metastasis, drug resistance, and immunosuppression [91,94,95,96]. In the past, ambiguity about the nomenclature of these vesicles resulted in usage of different terms like, exosomes, microvesicles, shedding vesicles, ectosomes, or microparticles. It is now agreed to label these vesicles as “extracellular vesicles”, which are further sub-categorized into exosomes and microvesicles, mainly based upon their site of origin. Vesicles generated in early endosomes are termed “exosomes” (~size 40–150 nm), while vesicles that are budded directly from the membranes are named ‘microvesicles’ (~size 100–1000 nm or more). It is now evident that cancer cells secrete higher amount of extracellular vesicles loaded with unique cargo proteins, miRs and lipids under hypoxic conditions [4,93,96,97,98]. For example, King et al. reported that under hypoxic conditions, breast cancer cells released an increased amount of exosomes, and that was dependent on HIF1α [97]. Furthermore, exosomes released by adipocytes under hypoxic conditions promoted lipogenesis in the recipient 3T3-L1 pre-adipocyte cells [99]. Similarly, hypoxia-resistant multiple myeloma cells secreted higher amount of exosomes compared to parental cells under normoxia or acute hypoxic conditions [95]. Ling et al. recently reported that exosomes derived from hypoxic oral squamous cell carcinoma cells (OSCC) increased the migration and invasiveness of OSCC cells in a HIF1α and HIF2α-dependent manner [100]. Hypoxic conditions also promoted microvesicles biogenesis dependent upon HIF1α and RAB22A protein expression, and these microvesicles significantly enhanced the formation of focal adhesions and invasiveness of naïve breast cancer cells [96]. Similarly, Berchem et al. recently reported that hypoxic tumor-derived microvesicles negatively regulate natural killer (NK) cell function through transfer of tumor growth factor (TGF)-β and miR23a [101].

We have recently reported that exosomes secreted by PCa cells under hypoxia promoted invasiveness, motility, epithelial-mesenchymal transition (EMT) and stemness in naïve PCa cells. These hypoxic exosomes also promoted a cancer-associated fibroblast phenotype in naïve normal prostate fibroblasts [93]. We also showed that hypoxic PCa exosomes were loaded with a higher number of unique proteins and signaling molecules (including matrix metalloproteinases, heat-shock proteins, TGFβ2, AKT, IL6 and TNF1α) compared to normoxic PCa exosomes. Subsequently, we found that hypoxic PCa exosomes were also loaded with a significantly higher amount of triglycerides, and that lipid metabolites played an important role in hypoxic PCa exosomes-induced invasiveness in naive PCa cells [4].

Several lipids such as BMP (*bis*-monoacylglycerolphosphate), ceramides, cholesterol, etc., are considered important in exosome biogenesis and loading [102]. Since lipid accumulation is one of the hallmarks of hypoxic cancer cells, it is plausible that increased accumulation of lipids also promotes extracellular vesicles biogenesis under hypoxic conditions. In this regard, our results highlighted the role of cellular lipids in the biogenesis of extracellular vesicles in PCa cells under both normoxic and hypoxic conditions. Moreover, the lipogenesis inhibitor silibinin significantly reduced the extracellular vesicles concentration as well as VEGF loading in extracellular vesicles [4]. These results advocate that extracellular vesicles synthesis as well as extracellular vesicles-regulated biological effects in the tumor microenvironment, could be targeted by targeting various pathways involved in lipid accumulation (described above).

## 4. Conclusions and Future Directions

Aberrant lipid metabolism plays an important role in PCa growth and progression. This altered metabolism includes lipogenesis and β-oxidation, which provides fuel and lipid signaling mediators to sustain growth and resistance to stressful environments. It is now clear that hypoxic cancer cells accumulate lipids that are important for their survival and proliferation, especially when reoxygenation occurs. Thus, metabolic and molecular pathways involved in lipid accumulation in hypoxic PCa cells, as well as lipid usage following hypoxia-reoxygenation, could be targeted therapeutically for better clinical management of PCa. Moreover, biomarkers and imaging techniques simultaneously measuring hypoxia and lipids in prostate tumors could offer better prognostic information about the disease.
